# Respiratory Source Control Using Surgical Masks With Nanofiber Media

**DOI:** 10.1093/annhyg/meu023

**Published:** 2014-04-15

**Authors:** Shaji D. Skaria, Gerald C. Smaldone

**Affiliations:** Division of Pulmonary, Critical Care & Sleep Medicine, Stony Brook University Medical Center, 100 Nicolls Road, HSC T-17–040, Stony Brook, NY 11794–8172, USA

**Keywords:** healthcare worker protection, mask comfort, mask compliance

## Abstract

**Background:**

Potentially infected individuals (‘source’) are sometimes encouraged to use face masks to reduce exposure of their infectious aerosols to others (‘receiver’). To improve compliance with Respiratory Source Control via face mask and therefore reduce receiver exposure, a mask should be comfortable and effective. We tested a novel face mask designed to improve breathability and filtration using nanofiber filtration.

**Methods:**

Using radiolabeled test aerosols and a calibrated exposure chamber simulating source to receiver interaction, facepiece function was measured with a life-like ventilated manikin model. Measurements included mask airflow resistance (pressure difference during breathing), filtration, (mask capture of exhaled radiolabeled test aerosols), and exposure (the transfer of ‘infectious’ aerosols from the ‘source’ to a ‘receiver’). Polydisperse aerosols were measured at the source with a mass median aerodynamic diameter of 0.95 µm. Approximately 90% of the particles were <2.0 µm. Tested facepieces included nanofiber prototype surgical masks, conventional surgical masks, and for comparison, an N95-class filtering facepiece respirator (commonly known as an ‘N95 respirator’). Airflow through and around conventional surgical face mask and nanofiber prototype face mask was visualized using Schlieren optical imaging.

**Results:**

Airflow resistance [ΔP, cmH_2_O] across sealed surgical masks (means: 0.1865 and 0.1791 cmH_2_O) approached that of the N95 (mean: 0.2664 cmH_2_O). The airflow resistance across the nanofiber face mask whether sealed or not sealed (0.0504 and 0.0311 cmH_2_O) was significantly reduced in comparison. In addition, ‘infected’ source airflow filtration and receiver exposure levels for nanofiber face masks placed on the source were comparable to that achieved with N95 placed on the source; 98.98% versus 82.68% and 0.0194 versus 0.0557, respectively. Compared to deflection within and around the conventional face masks, Schlieren optical imaging demonstrated enhanced airflow through the nanofiber mask.

**Conclusions:**

Substituting nanofiber for conventional filter media significantly reduced face mask airflow resistance directing more airflow through the face mask resulting in enhanced filtration. Respiratory source control efficacy similar to that achieved through the use of an N95 respirator worn by the source and decreased airflow resistance using nanofiber masks may improve compliance and reduce receiver exposure.

## INTRODUCTION

Reducing exposure to and emissions of infectious respiratory aerosols has become a major infection control issue with activity in the lay press and medical editorials increasing during each epidemic (Larson *et al.* 2010). Recommendations from regulatory agencies and consensus committees are largely based on limited *in vitro* data and often encourage facepieces to be worn by the presumed infected patient (‘source’) or the healthcare worker (‘receiver’) for the purpose of controlling the source of infection and protecting against infection. (Siegel *et al.* 2007; Larson *et al.* 2010) This article focuses on Respiratory Source Control (RSC) with the use of filtering facepieces, including face masks, commonly referred to as ‘surgical masks’, a novel nanofiber filtration face mask, and N95-class filtering facepiece respirators, commonly referred to as ‘N95 respirators’.

For any facepiece (face mask or respirator) to be effective it must be worn. In general, compliance wearing facepieces is less than ideal (Radonovich *et al.* 2009; Baig *et al.* 2010; Mitchell *et al.* 2012; Gosch *et al.* 2013). For example, numerous studies have demonstrated that healthcare workers are, in general, poorly compliant with respiratory protection guidelines, when an N95 respirator is recommended (Jefferson *et al.* 2009; Baig *et al.* 2010; Larson *et al.* 2010). In the community and for patient use, where wearers are unaccustomed to face mask use, there are significant social and comfort barriers to facepieces (Ferng *et al.* 2011).

Head and facial discomfort, in particular, the ‘heat’ inside a facepiece are often cited as reasons for noncompliance (Li *et al.* 2005; Radonovich *et al.* 2009; Shenal *et al.* 2012). This discomfort may correlate to the airflow resistance, measured as pressure differential (∆P), of the facepiece. Higher ∆Ps may cause increased work of breathing and/or encourage heat retention via deflection of warm exhaled breath within the facepiece. US military face mask specifications, in fact, correlate the airflow resistance (∆P) to a comfort scale in terms of perceived temperature within the face mask. Measurement of mask airflow resistance is included in the American Society for Testing and Materials (ASTM) standards for defining face mask material performance. Surgical masks, however, are commonly defined as ‘loose fitting’ as opposed to the intended proper fit of any respiratory protection device. These differences in fit and associated airflow leakage may impact ∆P, overall perceived comfort and compliance, and filtration efficacy. Because of the links between these factors, improvements in face mask design, focused on better breathability and greater filtration, may improve overall wearer compliance and source control efficacy.

Using an *in vitro* model, described in detail in recent studies (Diaz and Smaldone 2010; Mansour and Smaldone 2013), we compared the degree of exposure to a receiver from a potentially infected source with and without face masks composed of different materials including a new prototype face mask with lower airflow resistance. Our ultimate goal is a face mask that can be well tolerated by an infected source and provide significant receiver exposure reduction.

## METHODS

### Pressure differential (∆P)

To evaluate facepiece breathability, the pressure differential (ΔP) across each facepiece was measured. Figure 1 illustrates the setup. A Grass polygraph D.C. Driver Amplifier and recorder (Grass Instrument Co., Quincy, MA, USA; Model 7DAG) connected to a sensitive transducer (Setra Systems Inc. Transducer, MA, USA; Model 239ESS, Serial No. 24045) were used for resistance measurements. Attached to the transducer was a catheter inserted into the nostril of the same manikin used in the study conducted by Mansour and Smaldone (2013) [Resusci Anne CPR Manikin head (No. 310200; Laerdal Medical)]. The manikin was ventilated via a Harvard pump (Harvard Apparatus SN No. A52587; Millis, MA, USA) simulating a tidal breathing pattern (volume of 500ml, respiratory rate of 15 breaths min^−1^, and duty cycle of 0.5 s). The difference in air pressure inside the facepiece versus no facepiece during air exchange determined the pressure differential due to the facepiece (ΔP). Facepieces tested included an earloop face mask (SMnat, model No. GCFCXS; Crosstex International Inc, Hauppauge, NY, USA), a fitted face mask (Secure Fit^®^; model No. GCFCXUSF; Crosstex International Inc, Hauppauge, NY, USA), a prototype-fitted (Secure Fit) face mask with nanofiber filter media (PT), and a NIOSH-certified N95-class filtering facepiece respirator (N95, model No. 1860S size small; 3M, St Paul, MN, USA). Both the natural fit (SMnat) and fitted (SF) face masks had identical filtration materials that meet ASTM level 3 (most stringent) classification criteria. It is important to note that the only difference in the physical structure supporting the filter media of the natural fit versus the fitted and PT face masks was the extra metal band at the bottom of the fitted and PT masks. Masks were tested under both real-life conditions (placed on the face as intended, with potential to leak) and ‘true resistance’, e.g. ΔP was measured by sealing the facepiece to the manikin face with tape positioned along the edges to eliminate any leaks. Six samples of each facepiece were tested for each condition. As demonstrated in Fig. 2, each breath generated a bidirectional-tracing pattern (pressure swing); that represents inspiration (upward deflection) and expiration (downward deflection). One-half the value of the total swing estimated the unidirectional resistance (pressure differential) across the facepiece. Measurements were reported as *cm of H*_*2*_ *O*. (Note: ASTM standards report (∆P) in cm H_2_O cm^−2^, we did not fix or measure the area of the filter media.)

**1:**
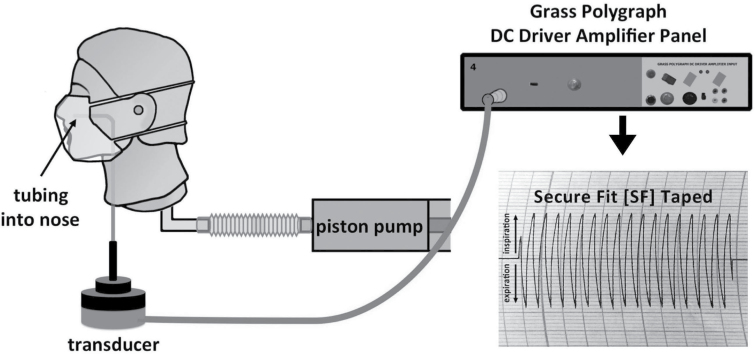
Schematic representation of setup used to measure pressure drop (ΔP) across a mask. Pressure was measured by a transducer attached to a catheter inserted into the nostril of a Resusci Anne CPR manikin. Ventilation provided by Harvard pump simulating tidal breathing. Tracings are shown.

**2:**
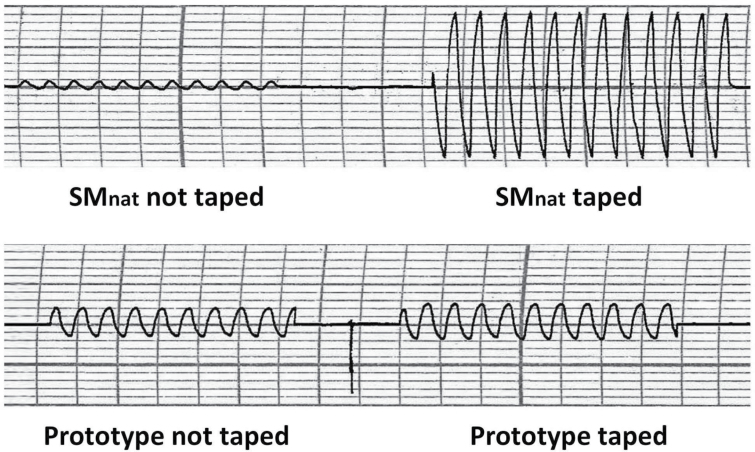
Pressure tracing of a surgical mask looped naturally around the ears (SMnat) and a prototype mask, constructed as a SecureFit™ Ultra Fitted mask with nanofiber filter media (PT) sealed and unsealed. Note difference in sealing SMnat versus PT indicating significant air leak present with loose fit mask versus PT mask.

#### Calibration

The transducer was calibrated prior to each experiment by applying a known pressure (Checkmate Pneumatic Verification Stand Model 1, Bourne Medical Systems, Inc., Riverside, CA, USA). Gain was adjusted such that a 50-mm deflection corresponded to 1cm H_2_O.

### Exposure chamber

A chamber detailed previously (Diaz and Smaldone 2010; Mansour and Smaldone 2013) was used to simulate ventilation in a hospital room (6 air exchanges h^−1^). Previous studies demonstrated that in this environment, filtration is the dominant mechanism of protection in RSC.(Diaz and Smaldone 2010) Therefore, for this study, we used a hospital room model to assess the effects of prototype (PT) face mask filtration compared with other facepieces worn on the source, on receiver exposure.

The chamber measured 5.7 ft in length × 5 ft width × 6.25 ft inches in height, with two Resusci Anne CPR manikins placed 3 ft apart. Each manikin was ventilated via a Harvard pump with the tidal breathing pattern. The source manikin represented the infected source and was connected to an AeroTech II nebulizer (three devices used in rotation; Biodex, Shirley, NY, USA) powered by an air tank at 50 PSIG, 10 l min^−1^. The nebulizer was filled with 3ml of 0.9% normal saline labeled with technetium-99m and run over 8min producing radioactive wet aerosols simulating contaminated particles exhaled during tidal breathing. In a previous study, using this chamber and cascade impaction, polydisperse aerosols were measured at the source with a mass median aerodynamic diameter of 0.95 µm. Approximately 90% of the particles were <2.0 µm (Mansour and Smaldone 2013).

### Mask airflow filtration

Airflow filtration, for purposes here, is the ability of the facepiece to capture aerosolized particles of the exhaled source breath and was reported as the percentage of radioactivity exhaled at the source that deposited on the facepiece. Test facepieces were placed only on the source manikin. We tested the facepieces’ abilities to filter under real-life conditions (unsealed) as well as by sealing them on the manikin face.

### Receiver exposure

Receiver exposure is the amount of aerosol exhaled by the source that was inhaled by the receiver. Receiver exposure was measured by placing a filter (model No. 041B0522; Pari, Starnberg, Germany) within the receiver manikin that captured all inhaled radioactive particles expressed as a fraction of activity exhaled by the source. For comparison purposes, we have calculated a relative receiver exposure factor defined as the ratio of MaxEx to actual exposure (Diaz and Smaldone 2010).

Devices with a range of sensitivities were used to measure radioactivity: a dose calibrator (10 μCi–10 mCi; Biodex, Shirley, NY, USA), a calibrated rate meter (1–10 μCi; Ludlum Measurements Inc., Sweetwater, TX, USA), or a calibrated microwell (0.01–1μCi; Kemble Instruments, Hamden, CT, USA).

### Schlieren optical imaging

Schlieren optical imaging relies on thermal differences in the air to refract a light beam in order to visualize airflows. While a human volunteer (wearing a SF, SMnat, or PT face mask) stood in front of a concave mirror, an illuminating light beam was directed at the center of the mirror to produce a real-time, visible image of the exhaled airflow as a thermal plume. Images were obtained with the subject breathing tidally or coughing (Tang *et al.* 2009; Tang, Nicolle, *et al.* 2011; Tang, Noakes, *et al.* 2011).

### Statistics

ΔP was reported as cmH_2_O, airflow filtration and receiver exposure data were reported as percent of nebulized particles (mean ± 95% confidence intervals [CI]). Group data were compared using 95% confidence intervals, and nonparametric comparisons of data were also performed using the Mann–Whitney test. Calculations were performed using GraphPad Prism v6.0 for Mac OS X (GraphPad Software, San Diego, CA, USA) and Microsoft Excel.

## RESULTS

### Pressure differential (ΔP)

Figure 3 illustrates ΔP across facepieces, sealed and unsealed. ΔP was significantly greater across the N95 respirator compared with all face masks; mean gradient of 0.2664 cmH_2_O (0.2426–0.2903 cmH_2_O). Sealing the N95 demonstrated no difference in ΔP, mean gradient of 0.2818 cmH_2_O (0.249–0.3146 cmH_2_O) indicating good fit without intentional sealing in our manikin model. Compared with unsealed N95, unsealed face masks (natural or fitted) demonstrated a dramatic reduction in airflow resistance; SMnat: mean of 0.00104 cmH_2_O (0.008562–0.1235 cmH_2_O) and SF mean of 0.08166 cmH_2_O (0.0496–0.1137 cmH_2_O), respectively. However, the ∆P significantly increased when the face masks were sealed to the face; SMnat mean of 0.1865 cmH_2_O (0.1762–0.1968 cmH_2_O) and SF mean of 0.1791 cmH_2_O (0.172–0.1861 cmH_2_O), respectively. Both the natural and fitted masks, therefore, relieve the pressure during breathing by allowing significant air leakage around the mask. When these leaks were sealed, the resistances approached that of the N95. ΔP for the prototype nanofiber mask (PT) was similar to the unsealed natural fit (SMnat) mask, but notably, sealing the mask minimally affected resistance (Fig. 3); unsealed PT mean of 0.03107 cmH_2_O (0.02466–0.03747 cmH_2_O) versus sealed PT of 0.05041 cmH_2_O (0.04449–0.05633 cmH_2_O). This observation indicated that air leaks around the prototype nanofiber mask were reduced compared with the natural and fitted masks and that the airflow resistance of the prototype mask was markedly less than that of the unsealed N95.

**3:**
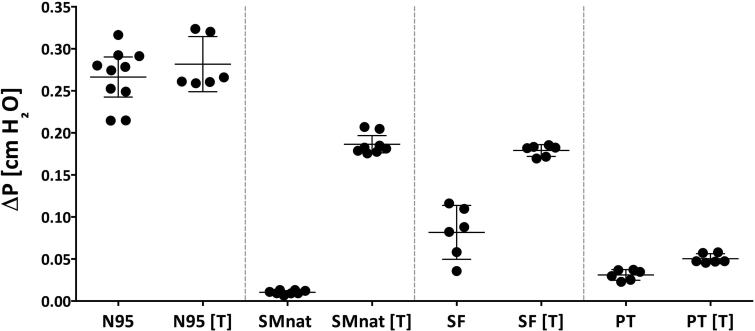
Pressure drop (ΔP ± CI) across N95 (3M respirator), SMnat (surgical mask looped naturally around the ears), SF (SecureFit™ Ultra Fitted surgical mask), and PT (SecureFit™ Ultra Fitted surgical mask with nanofiber filter media).

### Airflow filtration

Facepiece airflow filtration is shown on Fig. 4. For example, as expected, the N95 was able to filter 84.47% (95% CI: 82.93–86%) and when sealed 98.98% (95% CI: 95.2–102.8%). In comparison, SMnat and SF only filter 22.7% (16.03–29.38%) and 49.21% (95% CI: 43.32–55.1%), respectively. Because both face masks are made of identical textile material and differ only in terms of fit (the bottom metal band for the SF), it is the reduction of air leaking that results in a dramatic increase in filtration to 84.52% (95% CI: 77.6–91.44%). PT (a SF with nanofiber filter) was able to filter more efficiently than the commercial masks at 65.03% (60.47–69.58%) and when sealed 82.68% (80.57–84.78%).

**4:**
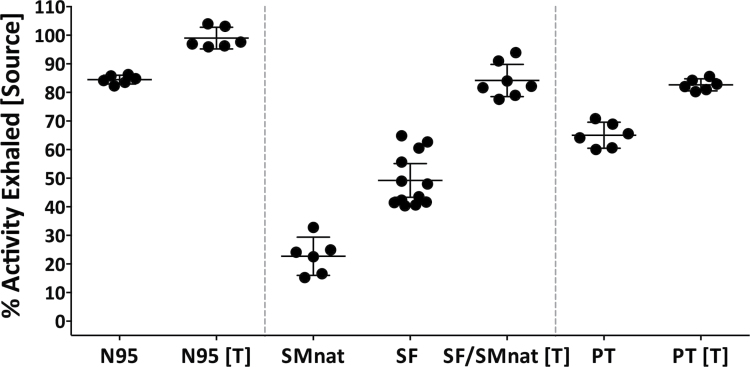
Airflow filtration efficiency (percent activity exhaled ± CI) with test mask placed on the source and Masks sealed and unsealed. N95 (3M respirator), SMnat (surgical mask looped naturally around the ears), SF (SecureFit™ Ultra Fitted surgical mask), and PT (SecureFit™ Ultra Fitted surgical mask with nanofiber filter media).

### Receiver exposure

Figure 5 demonstrates aerosol exposure to the receiver (Receiver Exposure). Data on the left represents ‘Maximum Exposure’ (Max Ex); the percent of nebulized particles captured on the filter in the receiver manikin when no facepiece is used on the source (Diaz and Smaldone 2010; Mansour and Smaldone 2013) For each facepiece placed on the Source, there was a significant reduction in receiver exposure (RSC). As expected, the most effective RSC facepiece was the N95. The natural fit face mask was least effective. All versions of the sealed SF face mask were as effective as the N95 with insignificant differences after sealing with tape (pNS).

**5:**
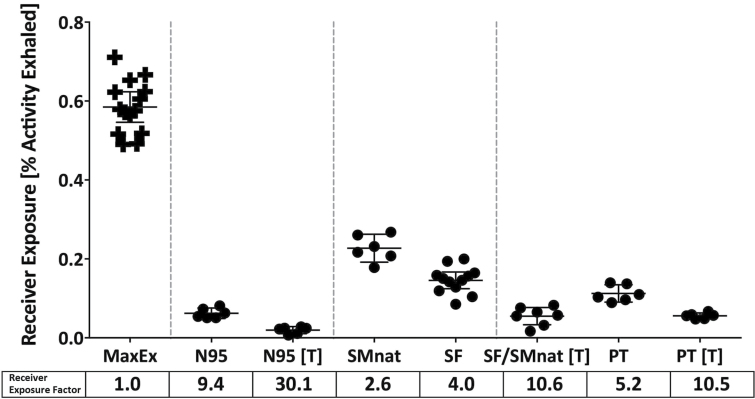
Receiver exposure: Defined as radioactivity inhaled by receiver manikin (as percent activity exhaled ± CI) when mask was placed on the source, masks tested are sealed and unsealed. N95 (3M respirator), SMnat (surgical mask looped naturally around the ears), SF (SecureFit™ Ultra Fitted surgical mask), and PT (SecureFit™ Ultra Fitted surgical mask with nanofiber filter media). Receiver exposure factor is defined as ratio of MaxEx to actual exposure, calculated for each mask type.

Receiver exposure factors are listed on the bottom of Fig. 5. In comparison to N95, face mask values were lower, but when sealed, values were similar to N95 (pNS). Receiver exposure factors for the unsealed prototype (PT) were slightly but significantly less than unsealed SF (*P* = 0.032). The unsealed PT was superior to the other unsealed face masks but less effective than the unsealed N95 (5.2 versus 9.4, *P* < 0.002).

### Schlieren optical imaging

Through imaging we were able to visualize varying airflow patterns created by the airflow deflection caused by each face mask. We found marked differences between the SF (Fig. 6) and PT masks (Fig. 7). As the individual breathes with a SF mask, obvious plumes of air are deflected within and around the mask. With the PT mask, more airflow is directed through the mask with an associated reduction in air flowing around the mask. This reduction of deflection enhances filtration. This visual effect mimics our objective measurements of airflow resistance.

**6:**
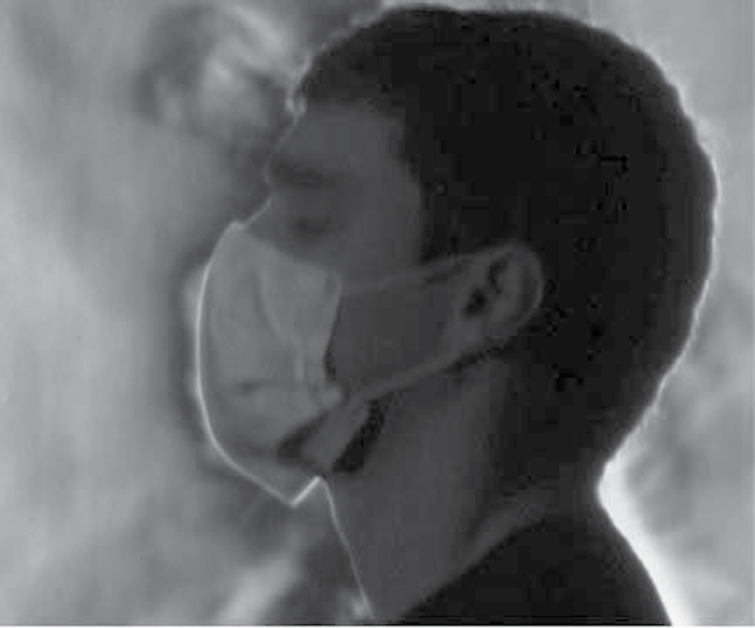
Schlieren optical imaging: Plumes of air during exhalation from healthy individual wearing SF mask, during tidal breathing. Air is leaking around the mask.

**7:**
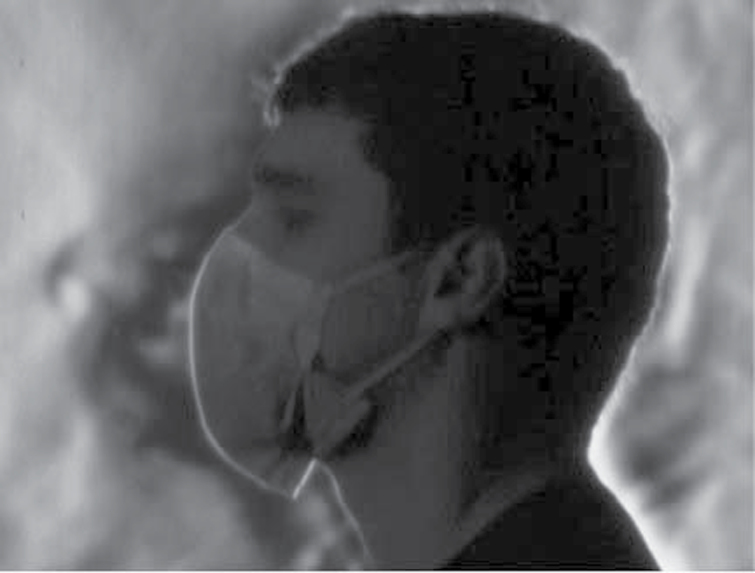
Schlieren optical imaging: Plumes of air from same individual as in Fig. 6 wearing PT mask. More air is exhaled through the mask with only a small amount of leak around the mask.

## DISCUSSION

Respiratory hygiene and cough etiquette are widely encouraged during severe influenza outbreaks and pandemic events such as during the 2009 H1N1 event as a means of respiratory source control; however, there is little data surrounding adherence to proper technique, overall compliance to, and clinical efficacy of these interventions. In clinical settings, patient masking is encouraged but only as tolerated for individual patients (CDC 2013). While several recent studies aim to show the contribution of mask use in reducing spread of infection, none isolate the impact of respiratory source control via index patient mask use and those focused on nonhealthcare mask use reveal suboptimal compliance. MacIntyre *et al.* (2009) showed overall mask (surgical and P2) compliance to be ≤50% among 290 adults in a household population study and estimated a potential 60–80% reduction in the daily risk of acquiring a respiratory infection with adherent mask use. Cowling *et al.* (2009) reported similar compliance rates in households with known infectious index patients. Canini *et al.* (2010) reported 75% of household study participants reporting discomfort while wearing a surgical mask (45% warmth, 33% breathing difficulties, 33% moisture). Other recent studies of facepiece compliance have suggested that the differences in resistance to airflow we have measured between N95 and our examples of surgical masks may not be detectable in real life. In a recent publication, Roberge and colleagues (2013) found that differences in airflow resistance among facepieces similar to those tested in our article were not detectable during exercise; however, all test subjects were well accustomed to using respiratory protection devices. When face masks are used for respiratory source control, wearers would likely include nonhealthcare professionals such as sick patients. For these novice users, small differences may be meaningful to improved compliance.

Surgical masks are classified as medical devices and have been used since the early 1900s in the healthcare setting to reduce transmission of infectious agents from worker to patient. Conversely, they are widely used to protect the healthcare worker from patient-generated aerosols and droplets (Belkin 1997). Commercial surgical masks (SMnat or SF) are designed with relatively large diameter filtration fibers (2–10 microns) responsible for maintaining a balance between filtration and pressure drop (Deniz 2012). They are made of melt-blown filter media inserted between two nonwoven fabrics. These mask layers, in total, must also pass ASTM standards for fluid protection, presumably to protect the wearer (healthcare worker) from patient-generated fluids.

Our main objective was to target source face mask compliance by developing a face mask that is comfortable to wear and effective in reducing infectious emissions from the wearer and thereby reducing receiver exposure. We found that nanofiber filter media lowered resistance to airflow while enhancing filtration of exhaled aerosols. The performance of the nanofiber prototype as a filter approached that of a commercial face mask sealed (taped) to the face. This effect was due to the enhanced flow through the nanofiber filter material as a consequence of nanofiber’s inherent reduced resistance to airflow.

We used ‘airflow resistance’ as an *in vitro* measure of comfort, and we recognize that our data are only an index of what may be important *in vivo*. For example, others have shown that compliance is related to the perception of humidity, heat, and high resistance noted by individuals wearing face masks or respirators (Li *et al.* 2005; Shenal *et al.* 2012). In addition, respirator use may be associated with increases in heart rate, skin temperature, perceived humidity, fatigue, breathing resistance, and overall discomfort compared to surgical masks (Li *et al.* 2005). But our data show that this difference may be largely attributable to the inherently loose fit of face masks rather than their material composition. While the tighter seal of any facepiece reduces air leakage and results in reduced receiver exposure, the resistance effects can reduce comfort and compliance. Airflow filtration, however, may be superior. When unsealed, our data indicate that all surgical masks have similar low resistance to breathing but standard masks do this because of leaks around the mask. Nanofiber masks may have similar low airflow resistance to breathing but will be better filters because of redirected flow.

Studies have shown that there is substantial leakage through the mask-nasal bridge interface to the upward direction and some downward leakage through the lower edges when wearing a surgical mask (Hui *et al.* 2012). As seen in our data, both commercial face masks demonstrated much lower resistance to breathing than respirators, but our data indicate that the drop in resistance is largely due to air leak around the face mask. The natural and fitted face masks have identical media differing only in the fit. When the leaks are eliminated, their airflow resistance approaches that of the N95. Similar observations were made for filtration. Our data, therefore, indicate that the major factors differentiating facepiece function are first, fit, e.g. N95 better than fitted face mask (SF) and fitted face mask better than natural fit (SMnat). The second factor is mask filtration. For existing commercial face masks, the only way to improve filtration and reduce receiver exposure is to improve the fit; however, improvements in fit result in increased mask airflow resistance because a higher proportion of the airstream is forced through the facepiece where the filter is located and not around it. The nanofiber media is more forgiving. The marked reduction in mask airflow resistance with the nanofiber media minimizes leaking around the mask and increases the chances that particles will go through the filter and be captured without the need for a perfect seal. Our data indicate that the improved fit of the SF design coupled with reduced airflow resistance of the nanofiber media reduces receiver exposure levels compared to face masks without a fitted design and nanofiber media.

Nanofibers are ~1 to 2 orders of magnitude smaller than melt-blown fibers and are produced by an electrospinning process (Grafe 2003) leading to a decrease in weight (0.02–0.5g m^−2^ versus 5–200g m^−2^ for melt-blown filter), increased surface area, and smaller micropores (Grafe 2003; Deniz 2012; Faccini *et al.* 2012). Contrary to the commercial face masks, the nanofiber prototype retained its low resistance, even when sealed to the face, as well as its filtration performance when unsealed. These differences in performance were supported qualitatively by the observations made during Schlieren optical imaging.

Our study was limited by the fact that it was an *in vitro* study. We believe, however, that *in vitro* studies can better define protocols for clinical trials. Real-life studies are required to assess mask effectiveness including wearer compliance and exposure protection. The choice of one mask versus another for planned clinical studies may be facilitated by objective *in vitro* measurements of function.

Compliance in wearing facepieces and wearing them properly, for the purpose of effective respiratory source control, is therefore important for both patients (or other potential sources) and susceptible persons around them (receivers). Our data indicate that receiver exposure to aerosols is reduced to a similar degree if the source wears either an N95 or the nanofiber prototype. Expecting a patient or other nonhealthcare professional to wear an N95 is impractical.

Our *in vitro* model does not address moisture loading of mask media and any clinical recommendation will need to test mask function over time. We are not aware of any formal regulatory guidelines, standards, or relevant published data in respect to long-term use of face masks.

## CONCLUSIONS

For the surgical face masks studied, incorporation of nanofiber filter media significantly reduced mask airflow resistance resulting in more of the exhaled air from the manikin passing through the face mask as opposed to bypassing the filter and going around the edges. Greater face mask compliance, and respiratory source control efficacy similar to that achieved through the use of an N95 respirator worn by the source, may be possible with continued improvements in face mask design.

## FUNDING

Partially funded by Cantel Medical Corp (1099556- 1-59631).
